# Effect of oral KETOPROFEN treatment in acute respiratory disease outbreaks in finishing pigs

**DOI:** 10.1186/s40813-018-0081-x

**Published:** 2018-03-06

**Authors:** Outi Hälli, Minna Haimi-Hakala, Tapio Laurila, Claudio Oliviero, Elina Viitasaari, Toomas Orro, Olli Peltoniemi, Mika Scheinin, Saija Sirén, Anna Valros, Mari Heinonen

**Affiliations:** 1 Faculty of Veterinary Medicine, University of Helsinki, Paroninkuja 20, 04920 Saarentaus, FI Finland; 2 Department of Clinical Veterinary Medicine, Estonian University of Life Sciences, Kreutzwaldi 62, 51014 Tartu, EE Estonia; 3 Faculty of Veterinary Medicine, University of Helsinki, P.O. Box 57, 00014 Helsinki, FI Finland; 4 Institute of Biomedicine, University of Turku, and Unit of Clinical Pharmacology, Turku University Hospital, 20014 Turku, FI Finland

**Keywords:** Behaviour, Daily weight gain, Acute phase proteins, *Actinobacillus pleuropneumoniae*, NSAID, Per os medication

## Abstract

**Background:**

Infection with respiratory pathogens can influence production as well as animal welfare. There is an economical and ethical need to treat pigs that suffer from respiratory diseases. Our aim was the evaluation of the possible effects of oral NSAID medication given in feed in acute outbreaks of respiratory disease in finishing pigs. The short- and long-term impact of NSAID dosing on clinical signs, daily weight gain, blood parameters and behaviour of growing pigs in herds with acute respiratory infections were evaluated. Four finishing pig farms suffering from acute outbreaks of respiratory disease were visited thrice after outbreak onset (DAY 0, DAY 3 and DAY 30). Pigs with the most severe clinical signs (*N* = 160) were selected as representative pigs for the herd condition. These pigs were blood sampled, weighed, evaluated clinically and their behaviour was observed. After the first visit, half of the pens (five pigs per pen in four pens totalling 20 representative pigs per herd, altogether 80 pigs in four herds) were treated with oral ketoprofen (target dose 3 mg/kg) mixed in feed for three days and the other half (80 pigs) with a placebo. In three of the herds, some pigs were treated also with antimicrobials, and in one herd the only pharmaceutical treatment was ketoprofen or placebo.

**Results:**

Compared to the placebo treatment, dosing of ketoprofen reduced sickness behaviour and lowered the rectal temperature of the pigs. Clinical signs, feed intake or blood parameters were not different between the treatment groups. Ketoprofen treatment was associated with somewhat reduced weight gain over the 30-day follow-up period. Concentration analysis of the *S*- and *R*-enantiomers of ketoprofen in serum samples collected on DAY 3 indicated successful oral drug administration.

**Conclusions:**

Ketoprofen mainly influenced the behaviour of the pigs, while it had no effect on recovery from respiratory clinical signs. However, the medication may have been started after the most severe clinical phase of the respiratory disease was over, and this delay might complicate the evaluation of treatment effects. Possible negative impact of ketoprofen on production parameters requires further evaluation.

## Background

Respiratory disease can influence production as well as animal welfare [1, 2]. For example, acute infection by one of the most common respiratory pathogens of pigs in Finland, *Actinobacillus pleuropneumoniae* (APP) is characterised by dyspnoea, cough, fever, reduced feed and water intake [3] and changes in white blood cell counts [3, 4].

Sickness behaviour is a consequence of inflammation. Typical sickness behaviour in pigs includes lethargy, reduced appetite, decreased motor activity and changes in thermal regulation. These changes help to preserve energy and to fight infection [5]. This response has been examined in experimental challenge studies. For example, pigs suffering from respiratory infection caused by porcine reproductive and respiratory (PRRS) virus spent more time lying in contact with another pig compared with non-infected controls [2]. In addition, PRRSv-inoculated pigs spent more time lying in ventral position and in contact with a penmate and less time in feeding compared to non-inoculated pigs [6].

Animals react to infections through an inflammatory response involving elevated levels of acute phase proteins in serum. In the pig, haptoglobin (Hp) is one of the most extensively investigated acute phase proteins. The concentration of Hp increases in serum within 48 h after infection [7, 8]. Respiratory infections can induce prominent increases in Hp levels as shown under experimental [9, 10] and clinical conditions [11, 12]. Another major acute phase protein in pig serum is amyloid A (SAA), which has shown the biggest difference in measured values between non-infected vs. infected animals of all investigated acute phase proteins [9, 13]. It has been suggested that acute phase proteins could be used as indicators of changes in animal health status or as aids in clinical diagnosis during infections as they are sensitive markers of infection [9].

There is an economical [14] and ethical need to treat pigs that suffer from respiratory diseases. Often, antimicrobial treatment is used. Recent evidence, however, indicates that non-steroidal anti-inflammatory drugs (NSAIDs) can also be beneficial in the treatment of respiratory diseases in pigs. NSAIDs (especially ketoprofen) have been reported to have a good ability to reduce fever [15–21] and at least to some extent to alleviate clinical signs [15–18, 20–22]. However, significant effects on blood parameters and growth have not been reported [15, 20, 21]. Evidence of the effects of NSAIDs on sickness behaviour is scarce and more information is needed; one recent study reported favourable impact of NSAID medication on sickness behaviour after lipopolysaccharide (LPS) administration [22].

The aim of our field study was to evaluate possible effects of NSAID medication administered in feed to finishing pigs during acute respiratory outbreaks in clinical conditions. Medication mixed in feed would be a practical alternative for medicating groups of diseased pigs instead of labour-intensive individual drug administration. However, sick animals usually eat less and hence it is important to evaluate whether medication mixed in feed could be successfully employed. We followed the short- and long-time impact of NSAID dosing on clinical signs, daily weight gain, blood parameters, Hp and SAA concentrations as well as on behaviour of pigs in groups of animals suffering from an acute outbreak of respiratory infection. Our assumption was that ketoprofen given in feed would alleviate clinical signs, reduce inflammation and decrease the behavioural effects of the disease, hence, improve the welfare of pigs and their daily weight gain.

## Methods

This study was a randomised, double-blinded, placebo-controlled clinical trial. It was also a substudy of a study focusing on respiratory diseases in pigs in Finland aiming to determine the main pathogens responsible for acute respiratory diseases [23]. Local practicing veterinarians and farmers were asked to inform the research group about acute outbreaks of respiratory clinical signs in Finnish finishing pig herds during 2011–2014. The herds were included in the bigger study according to the same inclusion criteria that were applied for the current substudy (see below).

In this study, clinical signs refer to signs detected during a basic clinical examination, excluding behavioural observations. Accordingly, by sickness behaviour we mean observations regarding posture or activity of pigs, as explained in the ethogram (see Table 1).

**Table 1:** The ethogram used in the behavioural analysis of representative pigs in herds with respiratory disease outbreaks

BEHAVIOUR	DEFINITION
*Posture*	
Walk	Moving all 4 legs.
Stand	Standing on 4 legs motionless.
Sit	Hindquarters touching floor.
Lie lateral	Lying on either side.
Lie sternal	Lying on the belly.
Lie alone	Lying on side or belly, without contact to other pigs.
*Activity*	
Active	Head up, alert while lying, sitting or standing (if cannot be identified as nosing, eating or drinking).
Eat	Head in the trough
Drink	Snout in contact with water nipple.
Nosing	Touching pen mate or pen structures with snout
Passive	Standing, sitting or lying motionless, head down, not alert.
Other	Invisible or none of the above.

After the first notification of an acute outbreak of respiratory disease, the research group personnel contacted the farm and ensured that the farm and the disease outbreak fulfilled the inclusion criteria: 1) the herd was rearing finishing pigs; 2) the herd showed acute respiratory signs e.g. cough, lowered appetite, apathy or mortality of animals; 3) the herd had either feeding arrangements that allowed all pigs in one pen to eat at the same time or automatic feeding devices that allowed individual feeding and medication for dosing of the oral medication; 4) the location of the herd had to be close enough (within 250 km driving distance) to the university clinic located in Mäntsälä, Southern Finland, for the research team to be able to organise the sampling and behavioural observations; and 5) the farmer was willing to participate. Altogether four farms were eligible for study enrolment.

### Farm visits and data collection

**Fig. 1:**
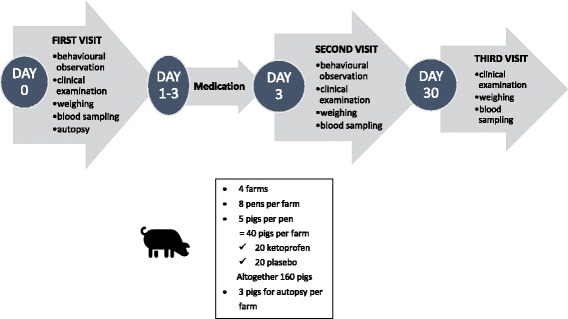
A scheme depicting the study design containing the farm visits, the treatment intervention and the numbers of study animals

A scheme depicting the study design containing the farm visits, the treatment intervention and the numbers of study animals is presented in Fig. 1. The research group first visited the farm for a baseline visit (DAY 0) within two days after the first notification of the acute outbreak. The farm visit started approximately 3.5 h before the midday feed was given to the pigs, allowing for behavioural observations two hours prior to feeding. Researchers first identified, after consulting the farm personnel, one compartment with the most profound clinical respiratory signs. During the first farm visit (DAY 0), three pigs per herd with acute respiratory clinical signs were selected for euthanasia and pathological examination to discern the pathogens involved in the outbreak. The lungs of these pigs were chilled and transported to the laboratory (Finnish Food Safety Authority Evira) for pathological, virological and bacteriological investigations to be begun on the next day.

From the selected compartment, eight pens containing the pigs with the most severe clinical signs were included in the study. Four pairs of two adjacent pens sharing one feeding trough were selected on the three farms using trough feeding. On the fourth farm using automated individual feeders, eight pens in one room were selected for sampling. Despite the shared trough, pigs in adjacent pens could not restrict the pigs in the other pen from approaching the feeding trough as there was a solid fence between the adjacent pens. The study pens housed an average of 11.3 (sd 1.4) pigs. From each pen, five representative pigs for the herd condition (altogether 40 pigs per farm) with the most severe clinical signs were numbered on their backs with marking spray from one to five. Half of the pens were randomly allocated for ketoprofen administration and the other half to the placebo treatment. Because of the shared feeding troughs, the pair of adjacent pens was always allocated to the same treatment. Each farm thus had 20 representative pigs divided into four pens receiving ketoprofen and another 20 pigs in four pens serving as a control group receiving treatment with placebo.

Two persons followed the behaviour of the representative pigs by direct observation with scan sampling every five minutes for two hours before the midday feeding equalling 24 observations per pig. The ethogram used is presented in Table 1.

After behavioural follow-up, the same 40 animals were evaluated clinically. Presence of clinical signs (YES/NO; tear staining, tail bitten, and any other clinical sign) were recorded. The farmer had been instructed to mark all pigs with colour that were seen coughing on DAY 0 before the researchers arrived. In addition, all pigs that were seen coughing during the farm visit in the selected compartment were recorded as having a cough. Rectal body temperature was measured and one EDTA and one serum blood sample was taken from the *vena jugularis* after catching the pig with a snout snare. An ear mark with an individual number was attached to the ear of these 40 pigs. Finally, each of these 40 pigs was weighed. Unfortunately, the scale used at farm #4 turned out to be unreliable and weighing results from this farm had to be discarded. Also, approximately half of DAY 30 weighing results from farm #1 are missing because of human error.

The second farm visit was carried out three days later (DAY 3). Behavioural and clinical evaluations were repeated, and the pigs were weighed, and blood was sampled (EDTA and serum samples). About 30 days (range 21–34 days) later (DAY 30), the farm was visited for the third time. The same pigs were evaluated clinically and weighed, and blood was sampled (only serum). Unfortunately, the data recordings made during the third farm visits were incomplete regarding clinical signs, leading to many missing values in the data.

### Medication

All pigs in the same pens with the representative pigs were given either ketoprofen 3 mg/kg or a placebo per os once a day for three days starting in the morning of DAY 1. The doses were calculated according to the number and weighing results of the representative pigs and the visually estimated weights of the rest of the pigs in each pen. The daily medications for each pen were dosed in small plastic bags. Herds 1–3 had long troughs, where all pigs fit to eat simultaneously. The farmers of these herds were instructed to mix the medication with small amounts (5–10 l) of regular pig feed and give the mixture by hand into the feeding trough once a day before the morning feeding. The farmer was instructed to follow the feeding during medication days and register any pigs that did not eat normally. In herd 4, the daily dose was given to the pigs through a sophisticated automated feeding system. Each pen had an automatic feeder, which identified each individual pig by its transponder device and delivered individual daily feed rations in several small portions during the day. The feeder had a dispenser capable of measuring small amounts of feed components. The dispenser measured, mixed and dosed the ketoprofen or placebo medication for each pig in the first feed portion in the morning. The feeder also measured the amount of feed eaten by each individual pig during the whole day.

The researchers had no possibility of deciding the antimicrobial medication given to the affected animals; only ketoprofen and placebo could be added in each herd. In herd #1, all pigs were treated with intramuscular injections of long-acting tetracycline (20 mg/kg) on DAYs 0, 2 and 4. In herd #2, intramuscular tetracycline injections (10 mg/kg) were given daily for 5 days to the pigs with clinical signs of respiratory disease. Unfortunately, the farmer had no exact records of the treated animals, but approximately 30% of all pigs in the room were treated and most of the representative pigs were likely to be included in the group of animals receiving tetracycline. In herd #3, all pigs were given intramuscular injections of tetracycline 3 and 1 days before the first farm visit (DAYs − 1 and − 3) and the treatment was continued per os (20 mg/kg) starting on DAY -1 for five days. In herd #4, no antibiotics were used because of mild clinical signs.

### Laboratory analyses

Blood samples were transported to the laboratory during the day of the farm visits and centrifuged (3000 rpm, 10 min) there. The sera were stored at − 18 °C until analysis. The EDTA samples were investigated on the day of sampling. The haemoglobin concentration (HGB), haematocrit (HCR), red blood cell count (RBC), white blood cell count (WBC), and platelet count (PLT) were measured for samples taken on DAY 0 and DAY 3 using an Animal Blood Counter Veterinary machine (ABX Diagnostics, Montpellier, France) in the Saari Laboratory, Faculty of Veterinary Medicine, Department of Production Animal Medicine, University of Helsinki.

For aerobic pathogen detection, the lung tissue samples were cultivated on bovine blood agar and incubated at 37 °C. In addition, for possible APP biotype 1 and *Haemophilus parasuis* isolation, the samples were cultivated on bovine blood agar with a *Staphylococcus aureus* streak and incubated under a 5% CO_2_ atmosphere at 37 °C. The small colonies showing enhanced growth around the *S. aureus* streak were isolated and confirmed by a positive Camp reaction. They were tested using multiplex PCR, which identified the species and APP serotypes 2, 5 and 6. The non-haemolytic NAD-dependent isolates with a negative CAMP reaction were further tested for *Haemophilus parasuis* using biochemical tests (oxidase, catalase, urease, fermentation of xylose, mannitol, inulin, trehalose and xylose supplemented with NAD and horse serum).

Serum Hp was analysed from samples taken on DAY 0, 3 and 30 using a modified haemoglobin-binding assay developed for cows [24], in which tetramethylbenzidine was used as a substrate [25] and 5 μl of sample volume (originally 20 μl). The assay was adapted for microtitration plates and optical densities of the wells were read at 450 nm using a spectrophotometer (Multiskan MS, Labsystems Oy, Vantaa, Finland). Pooled and lyophilized aliquots of porcine acute phase serum were used to create standard curves by serial dilutions. The standard curve range was 181–2900 mg/L. Samples with higher results than the standard range were diluted and re-assayed. The assay was calibrated using a porcine serum sample of known Hp concentration provided by the European Commission Concerted Action Project (number QLK5-CT-1999-0153). Serum SAA was analysed from samples taken on DAY 0, 3 and 30 with a commercial sandwich ELISA according to the manufacturer’s instructions for porcine serum (Phase SAA assay, Tridelta Development Ltd., Maynooth, Co. Kildare, Ireland).

Serum concentrations of both ketoprofen enantiomers (*S*- and *R*-ketoprofen) were determined for all DAY 3 samples from animals in the ketoprofen treatment group. In addition, eight samples taken on day 0 (before treatment) and eight from DAY 3 from the placebo-treated animals were analysed. Chiral high-performance liquid chromatography (HPLC) combined with UV detection was used for the quantitative analysis of ketoprofen enantiomers in pig serum. The analysis method was modified from a previously published description [26]. Samples were prepared by mixing 300 μl of serum, 50 μl of internal standard solution (*S*-(+)-naproxen, 100 μg/ml) and 650 μl of 0.4% formic acid in water:propanol (96:4). Solid-phase extraction cartridges were conditioned with 1.2 ml of 1% acetic acid in propanol and 1 ml of water. Samples (1 ml) were passed through the cartridges, which were then washed with 1.5 ml of 0.4% formic acid in water:propanol (96:4), followed by 1 ml of water. Solutes were eluted with 1.2 ml of 1% acetic acid in propanol. The solvent was evaporated to dryness in a stream of nitrogen. The residue was redissolved in mobile phase A and transferred into autosampler vials for HPLC analysis. The chromatography system consisted of a Waters Alliance 2695 Separations module and a Waters 2487 Dual λ absorbance detector. The analytical column was an Ultron ES-OVM chiral column (4.6 × 150 mm) preceded by a Ultron ES-OVM chiral guard column (4.0 × 10 mm), both from Shinwa Chemical Industries Ltd. (Kyoto, Japan). The mobile phase was an isocratic mixture of A: 16 mM phosphate buffer (pH 3.0) and B: acetonitrile (93:7). The flow rate was 1.0 ml/min. The UV detector was set at 254 nm. The chromatograms were processed using Empower 3 software (Waters). The linear concentration range was from 0.1 μg/ml to 20.0 μg/ml. Calibration curves were weighted by 1/x^2^ and yielded coefficients of determination (R^2^) of 0,997–1000 and 0,997–0,999 for *S*-ketoprofen and *R*-ketoprofen. The inter-assay accuracy of the quality control samples (at three different concentration levels, 0.3, 7.5 and 15.0 μg/ml) ranged from 94.9% to 104.4% for *S*-ketoprofen and from 94.1% to 103.7% for *R*-ketoprofen.

The samples from the 15 pigs (out of 20 sampled animals) having paired serum samples available after sample taking and processing, in sampling order, both from DAY 0 and DAY 30 were used for APP serology. APP antibodies were measured using two commercial test kits: IDEXX APP-ApxIV ELISA (IDEXX, Liebefeld-Bern, Switzerland) to detect antibodies against ApxIV toxin, which is produced by all known APP serotypes and IDvet ID Screen APP 2 indirect ELISA (IDvet, Grabels, France) to detect antibodies against LPS specific to APP serotype 2. Both tests were performed according to the manufacturer’s instructions. The absorbance results were interpreted as negative or unclear (score 0), or positive with scores ranging from 1 to 5. Seroconversion was defined as an increase in the score by at least one number, e.g. from negative to 1, or from 3 to 4, between DAY 0 and DAY 30. If both the first and the second sample showed the highest antibody level 5, the pig was also defined as seroconverted.

Similarly, the 15 animals from each herd having paired sera available from DAY 0 and DAY 30 were used for SIV serology. All blood samples were tested with influenza A antibody ELISA (ID Screen® Influenza A Antibody Competition, IdVet, Grabels, France) according to the instructions of the kit manufacturer. If at least one pig tested unclear or positive in influenza A ELISA on DAY 0 or 30, blood samples of that herd were further analysed using a haemagglutination inhibition (HI) test according to the operating procedure of the European Surveillance of Influenza in Swine with the antigens H1N1 (SW/Best/96), H1N2 (SW/Gent/7625/99) and H3N2 (SW/St. Oedenrode/96). All the antigens were provided by GD Animal Health Service (Deventer, NL). A sample was considered HI positive if the HI titre was ≥1:20. Seroconversion was defined as an increase in the HI titre between DAY 0 and DAY 30.

### Statistical analysis

A required sample size of 80 pigs per group was calculated for the main outcome (daily weight gain) with power 0.8 and confidence level 0.95 assuming equal variances and adjusted for clustering at pen level (assuming cluster size 11, intra cluster correlation 0.1 and coefficient of variation of cluster sizes of 0.1) and assuming a 100-g difference in daily weight gain between the treatment groups.

Descriptive statistics were calculated for daily weight gain, blood parameters, concentrations of *S*- and *R*-ketoprofen and for clinical signs and behaviour. An animal was used as the observational unit and results are presented as means and standard deviations (sd) for both treatment groups for all other data except behavioural variables. Mean occurrence (as a proportion of a total of 24 observations per pig) of each behaviour of the five pigs in each pen per observation day were calculated and pen mean was used in the statistical analyses. Because of different baseline levels of body temperature, weight, Hp and SAA concentrations on DAY 0 between the treatment groups, the changes in these variables between DAY 0 vs. DAY 3 within each treatment group were calculated by subtracting the value on DAY 0 from the value on DAY 3.

Descriptive statistics were compared within the treatment groups (ketoprofen/placebo) across different days by paired t-tests for body temperature, all blood parameters (except SAA values), weight and daily weight gain, by McNemar’s test for clinical signs, by a repeated measures general linear model for differences in behavioural variables, including farm as a fixed factor, and by Wilcoxon’s signed rank non-parametric test for SAA values. Effect of treatment on the magnitude of change in behaviour was tested with univariate models, including farm as fixed factor.

Drinking behaviour was very rare as was also ‘other behaviour´. These parameters were therefore not included when analysing the data. Due to technical errors during the observations, no records for activities were available for two of the pens on one farm.

Crude associations between outcome variables and treatment (ketoprofen/placebo) and other explanatory variables (sex) were evaluated using a liberal *p*-value (0.2) or strong suspicion of biological causal connection. Linear regression was used for that purpose for daily weight gain, body temperature and blood parameters excluding SAA values on DAY 0 and 3. Wilcoxon rank-sum non-parametric testing was used for SAA values on DAY 0 and 3. Logistic regression was used for variables related to clinical signs. Crude associations were not tested for behavioural variables.

Finally, multivariate models were built for the variables evaluated as significant in the crude association analysis and for the behavioural variables. A multilevel mixed-effects linear regression model was fitted for the outcomes weight and daily weigth gain, body temperature and change in body temperature as well as for the Hp and change in Hp and SAA concentrations, containing pen and farm as random intercepts and treatment and sex as fixed effects. Use of antimicrobial treatment in the herd to manage this respiratory outbreak (yes/no) was included as a fixed effect in the multilevel mixed-effects linear regression models for daily weight gain from DAY 0 to DAY 30 and for body temperature for DAY 3. In all other models, antimicrobial treatment did not remain significant and did not act as a confounder. A repeated measures general linear model with treatment and farm as fixed factors was fitted for behaviour variables. Because of marked variability of actualized interval of DAY 0 and DAY 30 herd visits, the number of days between the first and third herd visits was included in appropriate models. The level of significance was set to 0.05.

For brief model diagnostics, the basic assumptions of linear models were inspected with regard to the data structure and nature of the outcome variables. In addition, residuals were scrutinized. No serious breaches of the underlying assumptions were detected.

The results for Hp and SAA regarding third blood sampling (DAY 30) are presented only in the annex. The serum blood sample on DAY 30 was mainly taken for serology and acute phase proteins were analysed as the samples were readily available. However, it is unlikely that the ketoprofen medication used on DAY 1–3 had any effect on acute phase proteins on DAY 30.

Statistical analysis for all other outcomes than behaviour was made with STATA 14.2 program and the analysis of behavioural data was made with SPSS 21 statistical software.

## Results

Data consisted of 160 representative pigs for the herd condition from four farms: 75 (46.9%) castrated boars, 20 (20.5%) intact boars and 65 (40.6%) gilts, altogether 80 finishers in both the ketoprofen and placebo groups. In three herds, there were only castrated boars and females, which were distributed evenly in each herd. Intact boars were present on one farm, where they constituted half of the animals. All three sexes were divided evenly between the ketoprofen and placebo groups.

### Behaviour analysis

**Fig. 2:**
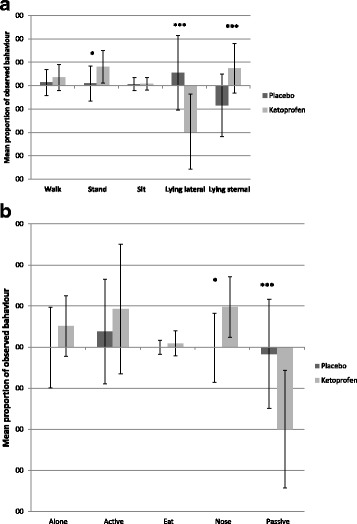
**a** Difference in mean (± sd) occurrence (as a proportion of a total of 24 observations per representative pig) of postures between DAY 0 and DAY 3. The pigs were given oral ketoprofen or placebo on DAY 1–3. Asterisks above bars indicate a significant difference in the magnitude of the change between placebo and ketoprofen pigs (* *p* < 0.05, *** *p* < 0.001). **b** Difference in mean (±sd) occurrence (as a proportion of a total of 24 observations per representative pig) of lying alone and behavioural activities between DAY 0 and DAY 3. The pigs were given oral ketoprofen or placebo on DAY 1–3. Asterisks above bars indicate a significant difference in the magnitude of the change between placebo and ketoprofen pigs (* p < 0.05, *** p < 0.001)

The results of the behavioural data for different treatment groups are presented in Table 2. Several behavioural differences are presented in the ketoprofen-treated pigs on DAY 3 as compared to DAY 0 based on repeated measures general linear model. On DAY 3, they were observed to stand, walk and lie more often on sternum and less often on flank and more often alone (not in contact with other pigs) than on DAY 0. In addition, the ketoprofen-treated pigs were more active and showed more nosing on DAY 3. Correspondingly, fewer observations of passive behaviour were recorded on DAY 3. In the placebo-treated pigs, the only observed change in behaviour from DAY 0 to DAY 3 was a decrease in lying on sternum. Figure 2a and b present the magnitude of these changes in the ketoprofen- and placebo-treated pigs.

**Table 2:** Behaviour of representative pigs in a group having a respiratory infection presented as proportion of observations mean ± sd out of 24 observations in two hours before treatment (DAY 0) and on the last day of treatment (DAY 3). The pigs were given ketoprofen or placebo on DAY 1–3

Behaviour	Treatment group	N of pens	DAY 0 Mean ± sd	N of pens	DAY 3 Mean ± sd
Lie flank	Placebo	16	0.32 ± 0.17^A^	16	0.37 ± 0.12^A^
	Ketoprofen	16	0.43 ± 0.19^a,B^	16	0.23 ± 0.11^b,B^
Lie sternum	Placebo	16	0.52 ± 0.17^a^	16	0.44 ± 0.11^b,A^
	Ketoprofen	16	0.43 ± 0.13^a^	16	0.51 ± 0.10^b,B^
Sit	Placebo	16	0.03 ± 0.02	16	0.04 ± 0.02
	Ketoprofen	16	0.02 ± 0.02	16	0.03 ± 0.02
Stand	Placebo	16	0.09 ± 0.07	16	0.10 ± 0.05^A^
	Ketoprofen	16	0.09 ± 0.08^a^	16	0.17 ± 0.08^b,B^
Walk	Placebo	16	0.04 ± 0.01	16	0.05 ± 0.05
	Ketoprofen	16	0.03 ± 0.03^a^	16	0.07 ± 0.04^b^
Lie alone	Placebo	16	0.09 ± 0.09	16	0.09 ± 0.06
	Ketoprofen	16	0.09 ± 0.10^a^	16	0.14 ± 0.11^b^
Active	Placebo	14	0.16 ± 0.10	14	0.20 ± 0.16
	Ketoprofen	14	0.11 ± 0.09^a^	14	0.21 ± 0.14^b^
Passive	Placebo	14	0.69 ± 0.14	14	0.67 ± 0.16
	Ketoprofen	14	0.80 ± 0.16^a^	14	0.60 ± 0.13^b^
Eat	Placebo	14	0.03 ± 0.03	14	0.03 ± 0.02
	Ketoprofen	14	0.03 ± 0.03	14	0.04 ± 0.04
Nosing	Placebo	14	0.10 ± 0.07	14	0.10 ± 0.07^A^
	Ketoprofen	14	0.05 ± 0.06^a^	14	0.15 ± 0.11^b,B^

^a,b^Values with different superscripts within the same row differ significantly, *p* ≤ 0.05

^A,B^ Values with different superscripts within the same column differ significantly, *p* ≤ 0.05

### Clinical signs and rectal temperature

The summary of clinical signs and rectal temperature of study animals during the clinical examination is presented in Table 3. Within the groups, the body temperature and number of coughing pigs decreased in both the ketoprofen- and placebo-treated pigs from the time of acute disease towards the end of the study when animals recovered from clinical disease. There was no treatment effect on any clinical signs based on results from single variable logistic regression models.

**Table 3:** Summary of clinical signs and rectal temperature of representative pigs in a group with respiratory infection

		DAY 0		DAY 3		DAY 30	
Variable	Treatment group						
		N	N (%) of animals	N	N (%) of animals	N	N (%) of animals
Cough	Placebo	80	9 (11.3)^a^	80	0 (0)^b^	50	0 (0)^b^
	Ketoprofen	80	7 (8.8)^a^	79	0 (0)^b^	50	0 (0)
Tear staining	Placebo	80	41 (51.2)	80	46 (56.3)	50	29 (58)
	Ketoprofen	80	42 (52.5)	79	42 (53.2)	50	34 (68)
Bitten tail	Placebo	80	20 (25)	80	13 (16.3)^b^	50	15 (30)^c^
	Ketoprofen	80	16 (20)	79	18 (22.8)	50	14 (28)
			°C mean ± sd		°C mean ± sd		°C mean ± sd
Rectal temperature	Placebo	80	39.8 ± 0.6^a^	80	39.4 ± 0.4^b,A^	80	39.3 ± 0.2^b^
	Ketoprofen	80	40.0 ± 0.8^a^	80	39.1 ± 0.4^b,B^	80	39.3 ± 0.3^c^

Pigs were given oral ketoprofen (3 mg/day) or a placebo during DAY 1–3. The clinical inspections were performed before treatment (DAY 0), on the last day of treatment (DAY 3) and on DAY 30

^a,b,c^Values with different superscripts within the same row differ significantly, p ≤ 0.05

^A,B^Values with different superscripts within the same column differ significantly, p = 0.01

According to the mixed model, rectal temperature tended to be somewhat lower (by 0.23 °C) on DAY 0 in the placebo group compared to the ketoprofen-treated group (*p* = 0.07). On DAY 3, pigs receiving the placebo had significantly higher (by 0.26 °C) rectal temperature than those treated with ketoprofen (*p* = 0.01). The pigs in the three herds with antimicrobial treatment did have 0.4 °C lower body temperature on DAY 3 than the pigs in the herd not receiving antibiotic treatment (*p* < 0.01). No difference was detected in body temperature on DAY 30. The rectal temperature change from DAY 0 to DAY 3 was affected by the ketoprofen treatment (*p* < 0.001). The body temperature of the placebo-treated pigs decreased less (on the average by 0.3 °C from DAY 0 to DAY 3) than that of ketoprofen-treated pigs (by 0.8 °C). There was no difference in temperature change between DAY 0 and 30 between the treatment groups.

### Acute phase proteins

Haptoglobin serum concentrations were measured for 77 ketoprofen-treated animals on DAY 0 and 75 for DAY 3 and for placebo-treated animals 79 and 77, respectively. Serum amyloid A concentrations were measured for 77 ketoprofen-treated animals on DAY 0 and 75 for DAY 3 and for placebo-treated animals 79 and 78, respectively.

**Fig. 3:**
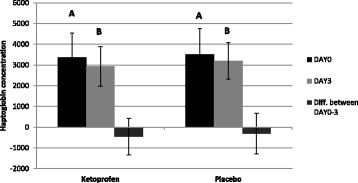
Haptoglobin concentrations (mg/l, mean and sd) for finishing pigs in herds having a respiratory disease outbreak. The pigs were given oral ketoprofen during DAY 1–3 and sampled before treatment (DAY 0) and on the last day of treatment (DAY 3). Bars marked with different letters (A, B) differ significantly from each other (p < 0.05), the comparison is valid only within treatment group (ketoprofen/placebo)

Hp serum concentrations were higher on DAY 0 compared to DAY 3 in both treatment groups (*p* < 0.01 for ketoprofen group and *p* = 0.01 for placebo group). Based on a mixed model, there was no difference in Hp concentrations between the treatment groups before treatment (DAY 0). The treatment was associated with Hp levels on DAY 3. Pigs in the placebo group had, on the average, 268 mg/l higher haptoglobin concentrations in serum than the pigs in the ketoprofen-treated group (p = 0.01). The Hp concentration change from DAY 0 to DAY 3 (calculated by subtracting Hp on DAY 0 from Hp on DAY 3) was not associated with the treatment. Figure 3 presents Hp concentrations of pigs in the ketoprofen- and placebo-treated groups.

**Fig. 4:**
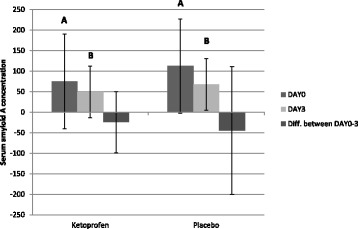
Serum amyloid A concentrations (mg/l, mean and sd) for finishing pigs in herds with a respiratory disease outbreak. The pigs were given oral ketoprofen or placebo during DAY 1–3 and sampled before treatment (DAY 0), on the last day of treatment (DAY 3) and on DAY 30. Bars marked with different letters differ significantly from each other (*p* < 0.01)

SAA concentrations were higher on DAY 0 compared to DAY 3 in both treatment groups (p < 0.01 for both treatment groups). Based on Wilcoxon’s rank-sum non-parametric test, there was no statistically significant difference between the treatment groups in SAA concentrations on DAY 0 or DAY3. The SAA concentration change from DAY 0 to DAY 3 (calculated by subtracting SAA on DAY 0 from SAA on DAY 3) was not affected by the treatment. Figure 4 presents SAA concentrations of pigs in both groups.

### Clinical blood parameters

Table 4 contains detailed results for complete blood count parameters. Treatment was not associated with any of the blood parameters analysed.

**Table 4:** Clinical blood parameters (mean and standard deviation, sd) of representative pigs in a group of finishing pigs with respiratory clinical signs

Variable	Treatment group		DAY 0		DAY 3
		N	(Mean ± sd)	N	(Mean ± sd)
WBC (10^9^)	Placebo	74	24.4 ± 6.4	74	25.1 ± 6.3
	Ketoprofen	75	23.1 ± 5.6	78	23.9 ± 5.3
HB (g/l)	Placebo	74	112.6 ± 10.0	74	110.9 ± 11.1
	Ketoprofen	75	110.5 ± 12.1	78	112.0 ± 11.0
HCT (%)	Placebo	74	34.5 ± 4.5	74	34.6 ± 3.9
	Ketoprofen	75	34.5 ± 4.2	78	34.7 ± 3.7
PLT (1000/μl)	Placebo	74	394.8 ± 127.3	74	411.6 ± 112.3
	Ketoprofen	75	368.0 ± 115.3^a^	78	414.3 ± 90.2^b^

The pigs were given oral ketoprofen (3 mg/kg) or placebo during DAY 1–3 and sampled before treatment (DAY 0) and on the last day of treatment (DAY 3)

White blood cell count = WBC, haemoglobin = HG, haematocrit = HCT, platelet count = PLT

^a,b^Values with different superscripts within the same row differ significantly, p ≤ 0.05

### Body weight, daily weight gain

The farmers reported three (2.5%) animals that did not eat normally during the medication days on the three farms having long troughs. On the fourth farm with the automated feeding system measuring the amount of feed eaten by each individual pig, all pigs included in the study ate the medicated feed portion by noon. The pigs in the placebo and ketoprofen groups ate similar and increasing amounts of feed from DAY 0 to DAY 3 on this farm.

Body weights were not statistically significantly different between the treatment groups on DAY 0 or DAY 3, even if the pigs in the placebo group tended to be somewhat lighter than those receiving ketoprofen. Based on mixed model, daily weight gain between DAY 0–3 was not significantly associated with the treatment, but pigs in the placebo group showed better daily weight gain (by 104 g per day) from DAY 0 to DAY 30 (*p* = 0.01) than the ketoprofen group. The pigs in the herds with antimicrobial treatment had 169 g less daily weight gain from DAY 0 to DAY 30 compared to the pigs in the herd not receiving antimicrobials (p = 0.01). However, antimicrobial treatment did not confound the ketoprofen treatment effect during the same time period. Table 5 shows all body weight-related results.

**Table 5:** Body weight and daily weight gain of representative pigs in a group of finishing pigs with respiratory clinical signs

Variable	Treatment group	N	DAY 0 Mean ± sd	N	DAY 3 Mean ± sd	N	DAY 30 Mean ± sd
Body weight, kg	Placebo	60	40.1 ± 7.1^a^	60	43.1 ± 7.5^a^	50	70.9 ± 9.5^b^
	Ketoprofen	60	43.4 ± 8.8^a^	60	46.3 ± 9.1^a^	50	71.1 ± 12.2^b^
			DAY0-DAY 3		DAY 0-DAY 30		
Daily weight gain, g	Placebo	60	1046 ± 719	50	992.5 ± 145^A^		
	Ketoprofen	60	1235 ± 721 ^a^	50	886.8 ± 197^b,B^		

The pigs were given oral ketoprofen (3 mg/kg) or placebo during DAY 1–3 and weighed before treatment (DAY 0), on the last day of treatment (DAY 3) and on DAY 30

^a,b^Values with different superscripts within the same row differ significantly, p ≤ 0.05

^A,B^Values with different superscripts within the same column differ significantly, p = 0.01

### Ketoprofen concentrations

**Fig. 5:**
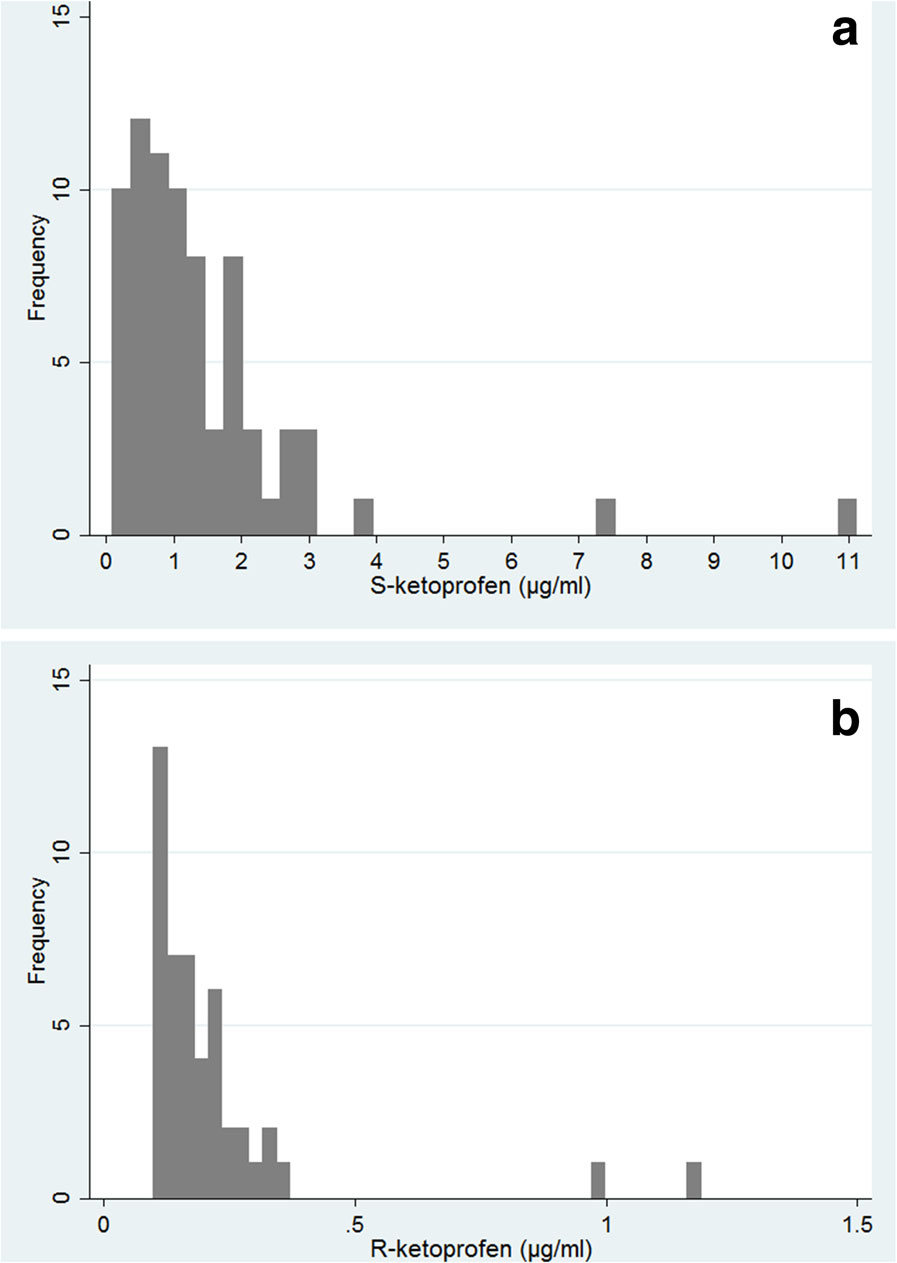
**a** Serum *S*-ketoprofen concentration frequency distribution (μg/ml) in samples (*n* = 79) taken on DAY 3 in ketoprofen treated finishing pigs in herds with a respiratory disease outbreak. **b** Serum *R*-ketoprofen concentration frequency distribution (μg/ml) in samples (n = 79) taken on DAY 3 in ketoprofen treated finishing pigs in herds with a respiratory disease outbreak

No samples collected from the placebo-treated pigs or collected from the ketoprofen-treated pigs before treatment start (baseline samples) contained detectable amounts of *S*- or *R*-ketoprofen. Serum *S*-ketoprofen concentrations were above the validated lower limit of quantification (LLOQ; 0.1 μg/ml) in 76 out of the 79 (96%) samples taken from ketoprofen-treated pigs on the last day of treatment. The remaining three pigs had serum *S*-ketoprofen concentrations just below the LLOQ. For *R*-ketoprofen, serum concentrations were above the LLOQ in 47 samples (60%). The mean (+sd) serum concentrations of *S*- and *R*-ketoprofen on DAY 3 were 1.41 ± 1.58 and 0.22 ± 0.20 μg/ml. The concentration frequency distributions of S- and R-ketoprofen on DAY 3 are presented in Fig. 5a and b. The blood samples for ketoprofen analysis were taken on DAY 3, on the average 324 ± 35 min after administration of the medicine mixed in feed.

### Pathogens involved in the respiratory infection

Based on the results from the autopsies and serology, all herds had an acute respiratory outbreak caused by APP and in one herd, also swine influenza virus may have been involved. At least one pig out of three autopsied per herd revealed an acute lung infection, where APP could be cultured and diagnosed as APP serotype 2. No other APP serotypes were discovered. In one individual animal, the pathological examination showed a mixed bacterial infection, where in addition to APP, *Pasteurella multocida* could be cultured. None of the autopsies revealed signs of other respiratory pathogens.

All four herds had pigs with seroconversion of APP antibodies in their paired serum samples. On the average, 67% and 45% of the pigs seroconverted between DAY 0 and DAY 30 based on either ApxIV toxin or APP2 LPS serological tests. In addition, in one herd, 13% of the animals had rising antibodies against SIV in their paired samples between DAY 0 and DAY 30.

## Discussion

The aim was to evaluate possible effects of NSAID medication administered in feed to finishing pigs during acute respiratory disease outbreaks in field conditions. Drug exposure was documented by concentration analysis in serum. Ketoprofen had the expected antipyretic effect and reduced behavioural signs of sickness. However, no statistically significant differences were noted between the ketoprofen- and placebo-treated animals in clinical signs, short-term weight gain or blood parameters. Unexpectedly, the animals of the placebo group showed better daily weight gain during the 30-day observation period after the medication than the ketoprofen-treated animals.

### Behaviour

The increase in activity and postures related to higher activity levels (standing, walking, lying on sternum) of the ketoprofen-treated pigs on DAY 3 compared to DAY 0 all indicate a decrease in behavioural signs of sickness [27], which was not apparent in the placebo-treated pigs. In our study, the pigs had only mild clinical signs and none of the animals was clearly dyspnoeic. Thus, we did not interpret sitting, or lying in a sternal position to be signs of pigs trying to ease their respiration. Instead, we interpreted lying in a sternal position as a sign of a more active behaviour compared to lying on the flank, which was further supported in the ketoprofen-treated pigs by increased nosing, which is a form of exploratory behaviour. Exploration has been reported to be reduced as a result of sickness [27]. The increase in lying alone in the ketoprofen group was interpreted as a sign of improved control of body temperature, as pigs are known to huddle when they experience a colder ambient temperature.

The only observed difference in frequency of behaviours on DAY 0 between treatment groups was in the lying in a flank position. We are unable to give any causal explanation for this difference. We suppose it to be coincidental especially because any other behaviours did not have baseline differences.

Altogether, the behavioural changes reported here are in line with changes seen in pigs experimentally infected with viral respiratory disease [2, 6]. Recently, ketoprofen has been shown to prevent the development of sickness behaviour, including depression caused by lipopolysaccharide-induced inflammation, which is in agreement with the observed higher activity levels observed in the current study [22].

### Clinical signs and rectal temperature

Before treatment, both groups had elevated rectal temperatures because they had been suffering from an acute respiratory infection for one or two days. On the last day of treatment, the body temperature had gone down in both treatment groups. The decline was, however, more pronounced in the ketoprofen group. This effect is understandable as ketoprofen has a good antipyretic effect in swine given in drinking water [15]. We show here that an antipyretic effect can also be achieved by giving ketoprofen in feed in clinical conditions.

We did not observe a treatment effect on any clinical signs (cough, tear staining, bitten tail) evaluated. Most previous studies reported an improvement in clinical signs on NSAID treated animals compared to the non-treated controls suffering from respiratory infection [14, 15]. In our study, some animals had already been treated with antimicrobial agents before the first visit, which may have lowered the severity of the clinical signs and made the possible differences in clinical signs pre- and post-medication smaller and thus less likely to be detected. Respiratory disease outbreaks were quite mild, as approximately only 10% of study pigs were coughing before the treatment. Furthermore, none of the pigs were coughing any longer at the second and third study visits, and dyspnea was not observed during the whole study period, which confirms the acute nature of the clinical respiratory disease in our study herds. On the other hand, as all pigs recovered swiftly, it may be that we missed the most acute phase of the disease episode. In case of an acute respiratory disease, the time elapsed after the beginning of the outbreak and the first herd visit may have been too long.

There were numerous missing values in the data regarding the clinical observations made on DAY 30, which may have led to observation bias. However, the missing values were distributed evenly to all herds and both treatment groups, which makes this bias unlikely.

### Haptoglobin and serum amyloid a

Regardless the treatment group, serum Hp concentration was elevated on DAY 0 compared to DAY 3 or DAY 30. Our samples were taken approximately two and five days post infection, even though there might be notable variation as we do not know the exact day of onset of clinical signs. As Hp reaches its peak concentration 2–3 days post infection and stays elevated up to 7 or more days post infection [10, 13, 28] it is likely that we had a good chance to detect elevated Hp serum concentrations. On all three sampling days, the Hp concentrations in both treatment groups were higher than the reference range (10–1310 mg/l) measured in a healthy boar herd [29]. A very similar range of Hp concentrations (2000–4000 mg/l) has been reported in pigs experimentally infected with APP or swine influenza virus [10, 13] and in pigs infected with several respiratory pathogens in clinical conditions [11]. However, it should be kept in mind that Hp concentration variation between herds could be notable depending on the overall health status and management of the herd, and Hp concentration comparisons between farms may not be very informative [30–32]. The magnitude of change in Hp concentrations from DAY 0 to DAY 3 was not affected by the treatment. Similar results of NSAID having no effect on Hp levels in endotoxin-challenged pigs have been obtained earlier [20].

SAA concentrations measured in specific pathogen free pigs are usually below 15 mg/l [11]. Compared to this reference value, SAA concentrations in this study before treatment in both treatment groups was clearly elevated and indicative of a positive acute phase response. Infected animals reach peak SAA concentrations within 1–2 days post infection and SAA seems to remain elevated at least 4 days post infection [10, 13, 28]. Our observed concentrations were in a similar range (40–60 mg/l) as those in swine influenza virus (SIV) infected pigs two days post infection [29]. In pigs experimentally infected with APP, even higher SAA concentrations (400–600 mg/l) were found 2–4 days post infection [10]. In co-infection with SIV and *Pasteurella multocida*, the peak level of SAA was observed as 155 μg/ml [13]. We did not find an effect of ketoprofen on SAA levels in pigs suffering from respiratory infection. Unfortunately, there is no previous knowledge regarding the effect of ketoprofen on SAA levels in infected pigs. In young calves, ketoprofen alone is able to decrease SAA concentrations after lipopolysaccaride challenge [33]. As the observed difference between the treatment groups in the change in SAA values from DAY 0 to DAY 3 was substantial, it might be that our study lacked sufficient power to detect a statistically significant treatment effect.

### Other blood parameters

The white blood cell count (WBC) was slightly elevated on DAY 0 and DAY 3 in pigs in both treatment groups compared to species specific reference values [34]. All other blood parameters in study pigs were within normal limits, as expected. There was no treatment effect on WBC which is in agreement with an older study where pigs were infected with APP [3]. The lack of treatment effect may be due to the overall mild clinical signs observed. On the other hand, very little is known about WBC alterations in pigs during respiratory disease or associated with NSAID treatment. It might be that they are not very sensitive markers of infection or effectiveness of NSAID treatment in pigs. Other researchers have also suspected that comparison to reference values is complicated by a number of factors, especially in pigs. For example, the values may not be applicable for modern pig breeds [35].

### Daily weight gain

There was no treatment effect on daily weight gain during the treatment from DAY 0 to DAY 3. In a previous study, no effect of ketoprofen on daily weight gain was seen during ten days of medication, when the drug was administered in drinking water to pigs suffering from porcine respiratory disease complex [15]. As already stated, the medication may have been started too late after the onset of an acute disease. If started earlier during the most acute phase of the disease, the medication may have had better possibilities to have the desired effect. As the observed difference between the treatment groups in weight gain from DAY 0 to DAY 3 was substantial, it might be that our study again lacked sufficient power to detect a statistically significant treatment effect.

We did observe approximately 100 g/day better average daily weight gain in the placebo group from DAY 0 to DAY 30 compared to the ketoprofen-treated group. Although unlikely, it cannot be ruled out that the three-day ketoprofen medication could have had a long-lasting effect on weight gain. The issue should be investigated further and biological explanations sought. The animals of the placebo group were by chance slightly smaller at the beginning of the trial than those allocated to the ketoprofen treatment, even if the difference was not statistically significant. We suspected that the observed difference in daily weight gain was at least to some extent due to compensatory growth of the smaller pigs in the placebo group. However, when this possible biological explanation was investigated further with analysis of covariance (results not shown), this was not the case.

No perceived difficulties occurred in administering the ketoprofen product per os mixed with regular pig feed. Pigs in three of the herds were on restrictive feeding where feed was available only at certain times and in limited amounts. Probably the restricted feeding made it easier to ascertain the intake of medicated feed, which was offered just before the regular feeding when the pigs were hungry. However, also in one of the herds with an automatic feeder, the pigs consumed the medicated feed with no problems. We did not observe treatment effect on appetite of pigs during the treatment, while the feed consumption increased steadily each day after DAY 0 (results not shown). In another study, NSAID treatment has been reported to lessen the decrease in feed consumption in infected pigs compared to non-medicated animals [3].

### Possible negative effects of NSAID medication

Even though NSAID medication may be helpful in inflammatory conditions, it should be kept in mind that negative effects have been reported in humans following NSAID consumption. Gastric ulceration is a well-known side-effect of NSAIDs in human medicine [36]. In the context of respiratory diseases, the frequency of severe bacterial infections after exposure to NSAIDs has been observed to be elevated in children [37]. Most studies on ketoprofen medication in pigs do not mention the possibility of negative effects. Such absence of information should not be interpreted as absence of possible drawbacks. However, in one study experimentally infected pigs were treated with NSAIDs, euthanized 48 h after the challenge and autopsied. None of the animals showed macroscopic kidney lesions or recent gastric ulcers [3]. In the current study, no negative effects were observed by the farm personnel during the three days of double-blinded medication with ketoprofen or placebo. However, it should be admitted that minor side effects, if present, might have gone unnoticed in the clinical setting. As already discussed earlier, 3-day treatment with ketoprofen was in our study associated with lower body weight gain during the subsequent 30-day observation period, but in the absence of a plausible biological explanation for such an adverse effect on growth, causality should not be assumed.

When per os medication is given to pigs in a group, the treatment is difficult, even impossible, to restrict only to certain animals in a group. In our study, ketoprofen (or placebo) was given to all animals in the same pen where the representative pigs were housed as this was considered to be the most feasible way to manage oral medication in most commercial piggeries. It is likely that some pigs received the medication even if they were healthy, thus exposing them to possible negative effects without any possible benefit. However, in case of respiratory diseases, the outbreak usually concerns the entire compartment. In addition to this, some animals might be subclinically infected and in need of medication. Thus, we suggest that making medication decisions on a pen-level would be accurate enough in the case of acute infectious respiratory disease in pigs.

### Ketoprofen concentrations

Medication mixed in feed of pigs comes with substantial risks of under- or overdosing. Our study shows that the pigs ate their ketoprofen dose voluntarily, because the total mean serum concentration was 1.6 μg/ml at six hours after feeding. In the ketoprofen concentration values of individual pigs, there was only one clearly higher (11.1 μg/ml) value. Based on this information, it is not likely that individual animals should have consumed the majority of medicated feed and possibly ingested significant overdoses of ketoprofen. Ketoprofen was administered as a racemic mixture of two enantiomers, *S*- and *R*-ketoprofen. Only *S*-ketoprofen is pharmacologically active. While both enantiomers have approximately similar pharmacokinetic properties in humans [38], they are handled in significantly different fashion in several animal species, including pigs [39]. Very marked chiral conversion of *R*-ketoprofen to *S*-ketoprofen has been observed, resulting in much faster clearance of *R*-ketoprofen compared to the *S*-form, and low exposure to *R*-ketoprofen [38, 40]. Our results are in line with these observations. The oral bioavailability of *S*-ketoprofen has been reported to be approximately 80%, when 3 mg/kg oral and intravenous doses have been compared in pigs [39]. The concentrations of *S*-ketoprofen in the serum samples collected approximately 6 h after administration of the drug mixed in feed were close to what has previously been reported after controlled oral administration of similar doses [39]. The maximum concentration of ketoprofen (measured as S- and R-ketoprofen or racemic ketoprofen) in serum is usually recorded 1–2 h after controlled oral administration by gavage [16, 41]. It has been reported that the mean racemic ketoprofen concentration in plasma was at least 1 μg/ml for about 10 h after oral administration at a dosage of 3 mg/kg in experimental settings, and this was theoretically considered as an effective dose [41]. Low total plasma racemic ketoprofen concentrations in pigs (0.1–2.09 μg/ml, depending on the dose given) have anti-inflammatory effects [17]. Very low half maximal inhibitory concentrations (IC_50_ 0.0003–0.003 μg/ml) of S-ketoprofen regarding inflammatory cytokine synthesis have been reported in the goat [42]. In this study, mean serum concentrations, derived from single blood samples, of S-ketoprofen and total racemic ketoprofen were 1.4 μg/ml and 1.55 μg/ml. Based on current, partly insufficient knowledge of required therapeutic levels for ketoprofen in pigs, the S-ketoprofen concentrations in our study should be estimated as adequate.

## Conclusions

The results indicate that ketoprofen administered in feed at approximately 3 mg/kg body weight reduced behavioural signs of sickness and had an antipyretic effect. As there were no effects of ketoprofen on clinical signs, feed intake or blood parameters, it can be assumed that the effect of ketoprofen mainly affected the welfare of the pigs, while the effect on recovery was less pronounced. However, the medication in this study was perhaps started only after the most severe clinical phase of the respiratory disease was over, and this delay might have hampered the evaluation of treatment effects. A possible adverse effect on production cannot be excluded, as the ketoprofen-treated animals showed lesser average growth over the 30-day observation period than the placebo-treated animals. Clinically significant drug exposure was achieved by administering ketoprofen per os mixed with regular pig feed in regular farm conditions in commercial herds.
